# *Mycobacterium ulcerans* Fails to Infect through Skin Abrasions in a Guinea Pig Infection Model: Implications for Transmission

**DOI:** 10.1371/journal.pntd.0002770

**Published:** 2014-04-10

**Authors:** Heather R. Williamson, Lydia Mosi, Robert Donnell, Maha Aqqad, Richard W. Merritt, Pamela L. C. Small

**Affiliations:** 1 University of Tennessee, Knoxville, Tennessee, United States of America; 2 University of Ghana, Accra, Ghana; 3 University of Tennessee Institute of Agriculture College of Veterinary Medicine, Knoxville, Tennessee, United States of America; 4 Michigan State University, East Lansing, Michigan, United States of America; University of California San Diego School of Medicine, United States of America

## Abstract

Transmission of *M. ulcerans*, the etiological agent of Buruli ulcer, from the environment to humans remains an enigma despite decades of research. Major transmission hypotheses propose 1) that *M. ulcerans* is acquired through an insect bite or 2) that bacteria enter an existing wound through exposure to a contaminated environment. In studies reported here, a guinea pig infection model was developed to determine whether Buruli ulcer could be produced through passive inoculation of *M. ulcerans* onto a superficial abrasion. The choice of an abrasion model was based on the fact that most bacterial pathogens infecting the skin are able to infect an open lesion, and that abrasions are extremely common in children. Our studies show that after a 90d infection period, an ulcer was present at intra-dermal injection sites of all seven animals infected, whereas topical application of *M. ulcerans* failed to establish an infection. *Mycobacterium ulcerans* was cultured from all injection sites whereas infected abrasion sites healed and were culture negative. A 14d experiment was conducted to determine how long organisms persisted after inoculation. *Mycobacterium ulcerans* was isolated from abrasions at one hour and 24 hours post infection, but cultures from later time points were negative. Abrasion sites were qPCR positive up to seven days post infection, but negative at later timepoints. In contrast, *M. ulcerans* DNA was detected at intra-dermal injection sites throughout the study. *M. ulcerans* was cultured from injection sites at each time point. These results suggest that injection of *M. ulcerans* into the skin greatly facilitates infection and lends support for the role of an invertebrate vector or other route of entry such as a puncture wound or deep laceration where bacteria would be contained within the lesion. Infection through passive inoculation into an existing abrasion appears a less likely route of entry.

## Author Summary

Buruli ulcer, a severe skin disease in West and Central Africa results in significant disability. The causative bacterium, *M. ulcerans* has been detected in many aquatic sources, but how bacteria enter the skin is an enigma. Two major hypotheses for infection are 1) that bacteria are injected into the skin through the bite of an aquatic insect, or 2) that bacteria enter open wounds on a person's body. In this study, we use a guinea pig infection model to evaluate whether application of *M. ulcerans* to an open abrasion produces Buruli ulcer. Our results show that despite topical application of very large numbers of *M. ulcerans*, we are unable to produce infection in open abrasions. These results are extremely surprising because most bacteria such as *Staphylococcus* or *Streptococcus* can readily infect abrasions. In contrast, intra-dermal injection of *M. ulcerans* into the skin of guinea pigs consistently produced an ulcer. Our studies are the first to explore the route of infection of *M. ulcerans* in an experimental model. These results suggest that Buruli ulcer is not likely to be due to passive entry of bacteria into pre-existing abrasions and supports the role of biting invertebrates, puncture wounds, or lacerations as a requirement for infection.

## Introduction

Buruli ulcer, a severe cutaneous infection caused by the environmental pathogen *Mycobacterium ulcerans* is a major cause of morbidity in West and Central Africa [1], [2]. The disease begins with a painless nodule that can lead to severe ulceration. Though mortality is low, morbidity is extremely high. In 1998, the World Health Organization declared Buruli ulcer a neglected tropical disease and established the Global Buruli Ulcer Initiative focused on prevention, awareness, and improved treatment options for those suffering from this disease. The transmission of *M. ulcerans* from the environment to humans is a central enigma in *M. ulcerans* research [1]. Infection has been consistently linked with exposure to aquatic environments, but the exact mode of transmission is unknown [3]. Person-to-person transmission is extremely rare [4].

Several routes of transmission have been proposed including: transmission via insect vectors [5]–[7]; direct contact with contaminated vegetation [1], [8], [9]; aerosol [9], [10] or entrance of the bacterium through a preexisting wound following environmental exposure [1], [9]. Of these, transmission by inoculation into pre-existing wounds or inoculation by the bite of an invertebrate vector has received the greatest attention. Superficial skin lesions are extremely common among children in the tropics. Abrasions and small open lesions are ubiquitous in children, but lacerations and puncture wounds also represent sites where bacteria could be introduced. Some of these hypotheses are supported by detection of *M. ulcerans* DNA in environmental samples [11]–[13].

Laboratory studies confirm that *M. ulcerans* survives in many invertebrate species and in one case transmission from an invertebrate vector to a mouse has been demonstrated experimentally under laboratory conditions [5]. However, invertebrate species implicated in transmission in West Africa are not hematophagous. The importance of invertebrates in maintaining aquatic food webs was summarized in a review of transmission by Merritt, et al [1], but the authors suggest that the role of invertebrates as vectors remains unclear. A study by Benbow et al, 2008 [14] also casts doubt on invertebrate vectors. That study did not, however, include sampling sites from a historically non-endemic region. More recently Benbow et al, 2013 [15] compared results from detection of *M. ulcerans* DNA in invertebrates from Buruli ulcer endemic sites with results from invertebrates from the Volta region in Ghana where Buruli ulcer is rarely encountered, and found that *M. ulcerans* DNA was not detected in invertebrates collected in the Volta region.

Cutaneous infections in pre-existing abrasions caused by waterborne pathogens are being recognized with increasing frequency [16]. *Aeromonas hydrophila*, *Pseudomonas aeruginosa*, and *M. fortuitum* are often associated with trauma and water exposure [17]–[20]. *M. marinum*, whose genome shares 98% sequence 16S similarity to *M. ulcerans*, is a pathogen of fish which can cause cutaneous infection in humans through contact with contaminated water [18], [21].

Although there have been a few reports of *M. ulcerans* developing at the site of a previous wound [22] or insect bite [23], there is no epidemiological data supporting this as a frequent mode of transmission. However, the incubation period between infection and disease is usually at least five weeks, and can be over six months [1], [24]. If the time span is several months, a patient might not remember the presence of a previous abrasion at the site of Buruli ulcer.

The objective of this study was to develop an infection model to determine the effect of the route of inoculation on the development of disease. For this model, we used hairless Hartley guinea pigs (*Cavia porcellus*). Guinea pigs are often used in cutaneous infection models because guinea pig skin is structurally and immunologically more similar to human skin than murine skin [25]. Both human and guinea pig skin have a thick fat layer that provides an ideal environment for the replication of mycobacteria. Buruli ulcer has been experimentally induced in guinea pigs by intradermal inoculation, a method that reproduces similar clinical manifestation and pathology to that produced in human skin [26]–[28].

Abrasions were made on the backs of hairless Hartley guinea pigs with a steel brush and the animals were exposed either topically to *M. ulcerans* or through intra-dermal injection. Additionally, two guinea pigs were topically infected with *Staphylococcus aureus* as a positive control for skin infection and inflammation. Results from these studies show that injection of *M. ulcerans* greatly facilitates the induction of Buruli ulcer, and suggest that entrance of organisms through a superficial skin abrasion is an unlikely route of transmission.

## Materials and Methods

### Bacterial strains and growth conditions

A well-characterized clinical isolate, *M. ulcerans* 1615 was used in all studies [26]. *M. ulcerans* 1615 were grown in Middlebrook 7H9 liquid media and on Middlebrook 7H10 agar supplemented with 10% oleic acid-albumin-dextrose enrichment (OADC) and incubated at 30°C to reach exponential phase of growth. Bacterial viability was validated by staining using a cell viability assay kit (Promega, Madison, WI, USA). *Staphylococcus aureus* 502a (ATCC# 27217) was obtained from American Type Culture Collection, cultured on nutrient agar and incubated at 37°C. This strain of *S. aureus* was isolated from the nares of a nurse, and described in ATCC as being coagulase positive, penicillin sensitive, and sensitive to 10 mcg of tetracycline.

### Animals

Male and female Hartley Hairless guinea pigs weighing between 250–300 g were used for inoculation experiments. Seven subjects were used in the first experiment reported here, and 12 for the experiment in which a time course was performed. The initial animals were housed in Walters Life Sciences Animal facility in separate cages.

### Experimental infection

Guinea pigs were transferred to the procedure room and placed under anesthesia of 2% isofluorane for approximately 4 minutes. Guinea pigs were maintained on continuous flow of oxygen and isofluorane for all procedures including abrasions, inoculation or injection. A steel brush was used to make skin abrasions on the backs of guinea pigs until blood was drawn (Figure S1). Immediately following this, twenty microliters of 10^4^ and 10^8^ *M. ulcerans* were dropped onto duplicate abraded areas in a 20 µL volume. Controls sites included: 1) a negative control where sterile media was dropped onto abraded skin, 2) a negative control where *M. ulcerans* was spread onto unabraded skin (Figure S1), 3) a positive control where 200 µL containing 10^6^ *M. ulcerans* 1615 was injected into the hind flank using a 25- gauge needle as previously described [26], [28], and 4) a positive control in which *S. aureus* (10^8^ CFU/ML in a 20 µL volume) was introduced to abraded areas of two guinea pigs in duplicate as a positive control for infection and inflammation. This inoculum for *S. aureus* was chosen based upon a published study showing that this concentration induced pathology 24-hours post infection in a dermal guinea pig model [29].

Infected guinea pigs were allowed to recover from anesthesia and transported to individual housing quarters when ambulation was restored. Guinea pigs were monitored by an attending veterinarian and animal care facility staff during the procedure, and daily throughout the study. Buprenophrine (0.5 mg/kg) was available to manage pain. However, *M. ulcerans* infections are painless and the duration of infection with *S. aureus* was short enough that pain management was not necessary.

Due to concerns of potential pain associated with *S. aureus* infection, guinea pigs infected with *S. aureus* were sacrificed 24-hours post infection. In an initial experiment, guinea pigs infected with *M. ulcerans* were sacrificed at 90 days p.i. In a subsequent experiment, animals were sacrificed at 1 hour, 24 hours, 48 hours, 7 days, and 14 days. Tissues were divided into quarters for culture, DNA extraction and qPCR, lipid analysis, or histopathology. Duplicate abraded sites exposed to *M. ulcerans* were divided in half and one of these tissue divisions was randomly used for one of the four analyses.

### Histology

Skin specimens for histology were routinely fixed in 10% buffered neutral formalin, paraffin embedded, and sectioned at 5 microns. Serial sections were stained with Modified Kenyon's stain and Hematoxylin–Eosin stains as described [26], [28].

### Isolation of *M. ulcerans* from tissue

Skin specimens were decontaminated using the modified Petroff's method as previously described [30]. Briefly, two milliliters of 4% NaOH was incubated with approximately 2 grams guinea pig tissue for 15 min, followed by centrifugation and decanting of the supernatant. Fifteen milliliters of sterile saline were added to the tissue pellet, and centrifuged at 3,000× *g* for 15 minutes. The supernatant was decanted and the decontaminated tissue was plated onto M7H10 agar plates supplemented with 10% OADC supplement and Lowenstein Jenson plates. All media was incubated at 30°C and observed weekly for signs of growth. Tissues infected with *S. aureus* were plated onto nutrient agar and incubated at 37°C for recovery of bacteria.

### DNA extraction and molecular analysis

Approximately 1 gram of tissue was lysed mechanically and chemically by bead beating in lysis solution for 15 minutes followed by incubation at 65°c for 20 minutes. Tissues were centrifuged for 2 minute at 5600×g and the supernatant was added to potassium acetate and incubated for 1 hour at −20°C. Samples were centrifuged for 30 minutes at 5600×g and the supernatant mixed with guanidine hydrochloride solution and added to a MOBIO spin filter. Each spin filter was centrifuged three times for 2 minutes at 5600×g with the flow through discarded each time. The spin filter was washed with wash solution and ethanol and the spin filter was allowed to dry by centrifugation at 5600×g for 5 minutes. DNA was eluted using elution solution and centrifugation and the resulting DNA was subjected to quantitative PCR analysis targeting the enoyl reductase domain of the plasmid responsible for mycolactone production as previously described [31].

### Ethics statement

The University of Tennessee Institutional Animal Care and Use Committee (IACUC) approved all procedures and protocols carried out in this study under IACUC protocol #1832. The University of Tennessee policies for animal care and use encompass regulations of the Animal Welfare Act as amended (Public Law 99–198 – The Improved Standard for Laboratory Animals Act), Guide for the Care and Use of Laboratory Animals (8th Ed.) and The Guide for the Care and Use of Agricultural Animals in Research and Teaching.

## Results

### *M. ulcerans* fails to establish infection through an abrasion during a 90d infection period

In order to determine whether *M. ulcerans* could establish infection through an open wound, abrasions were made on the backs of Hairless Hartley guinea pigs and *M. ulcerans* in Middlebrook 7H9/OADC media was dropped on the open abrasions. An intra-dermal injection was included as a positive control. A wheal was apparent following injection confirming the intra-dermal location of injection (Figure S1). Guinea pigs were sacrificed at 90 d p.i.

**Figure 1:**
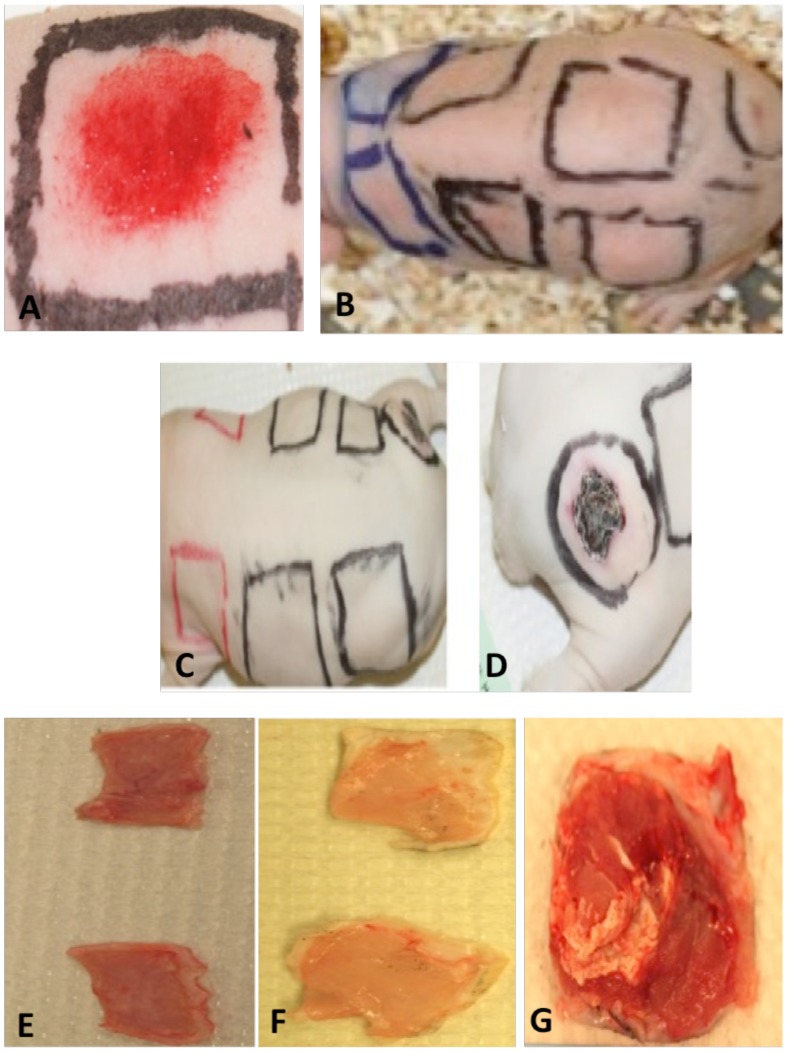
Gross pathology associated with *M. ulcerans* infection. A) Uninfected abrasion control 5 minutes p.i. B) Guinea pig 5 d p.i. showing healed abrasion sites and erythema at injection site. C) Healed abrasion sites 90 d p.i. D) Ulceration at injection site 90 d p.i. E) Ventral side of uninfected abrasion control site 90 d p.i. F) Ventral side of abrasion site where 10^8^ *M. ulcerans* was applied 90 d p.i. G) Ventral side of *M. ulcerans* injection site 90 d p.i.

No gross pathology was detected at any of the abrasion sites. All of the abrasion sites healed within the first week p.i., and remained healed throughout the remainder of the study (Figure 1B and 1C). Lesions developed at the injection site within the first two weeks and ulcers or plaques were present at the injection sites on all subjects at the end of the 90-day study period (Figure 1D). The ventral side of dissected lesions from the injection sites developed a typical “bacon-fat” appearance with evidence of necrosis and hemorrhage typical of Buruli ulcer [32] (Figure 1G) The ventral side of unabraded control tissues (1E) was identical to that of healed infected abrasions (1F).

**Figure 2:**
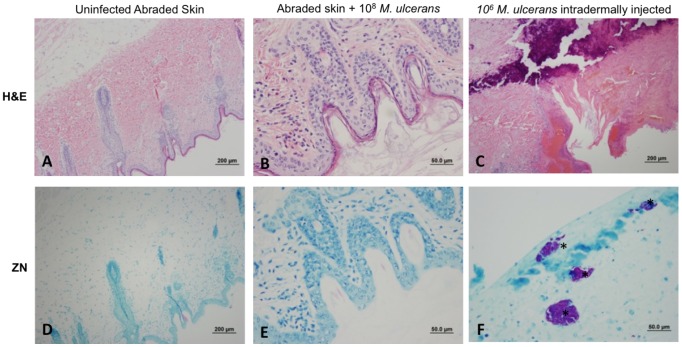
Histopathology of control and *M. ulcerans* infected tissues 90 days p.i. H&E (top panels) and Ziehl-Neelsen (bottom panels) stained sections. A) Uninfected abraded control (H&E) D) uninfected abraded control (ZN); B) Infected abrasion (H&E); E) Infected abrasion (ZN); C) Infected injection site (H&E); F) Infected injection site (ZN) Asterisks (*) indicate acid fast bacteria.

Histologically the abrasion sites where *M. ulcerans* was applied appeared normal (Figure 2B) and were identical to those of negative controls (Figure 2A). No acid-fast bacteria were detected in uninfected abraded skin (2D) or in infected abraded skin (2E).

In contrast, extensive microscopic pathology was observed in lesions formed by injection *of M. ulcerans* (2C). Extensive acellular necrotic foci, edema and calcification were characteristic features of these lesions. Hyperplasia was present at the site of inoculation and infiltration of inflammatory cells could often be detected at the edge of the necrotic center. Severe necrosis of subcutaneous adipose tissue was evident and in some animals necrosis extended to muscle tissue. Erosion of blood vessels was often evident as previously described [33]. Clusters of extracellular acid-fast bacilli were found adjacent to large areas of necrosis (2F).

*Mycobacterium ulcerans* was cultured from all of the guinea pig injection sites, whereas mycobacteria were not recovered from any abrasion site despite the high inoculum initially applied (Table 1). *M*ycobacterium *ulcerans* was detected by quantitative PCR in 7/7 injection sites with concentrations ranging from 7.25×10^5^ to 3.87×10^8^ genome units/sample (Table 1 and Figure S2). *Mycobacterium ulcerans* DNA was not detected in tissue from low dose infections from abrasions (10^4^), but *M. ulcerans* DNA was detected in 2/7 abrasion sites where 10^8^ bacteria were applied to abrasions (Table 1). Control tissues were negative for *M. ulcerans* DNA (Table 1 and Figure S2).

**Table 1:** Detection of *M. ulcerans* following topical infection of abrasions (10^8^ M. *ulcerans*) or by injection (10^6^ *M. ulcerans*) 90d p.i.

*M. ulcerans* Infection 90 d p.i.				
Treatment	Culture	AFB	Histopathology	qPCR GU/sample
Abraded+10^4^ *M. ulcerans*	0/7	0/7	0/7	0/7
Abraded+10^8^ *M. ulcerans*	0/7	0/7	0/7	2/7*
Injection 10^6^ *M. ulcerans*	7/7	7/7	7/7	7/7

*qPCR results are from 2 positive guinea pigs with qPCR values of 6.27E+03 and 2.10E+05 GU/sample.

*M. ulcerans* DNA was not detected in any other abraded guinea pig tissue at the 90 day timepoint.

### Transient colonization of an abrasion with *M. ulcerans*

Results from our first experiment showed that *M. ulcerans* was unable to establish an infection through an abrasion, but did not provide temporal data regarding colonization. To determine how long *M. ulcerans* remained in tissue following infection, an experiment was conducted to monitor the presence of *M. ulcerans* at 1 h, 24 h, 48 h, 7 d, and 14 d p.i. As in the previously described experiment, abrasions were made in the back skin of guinea pigs to a depth that bleeding was evident. For infection through injection, the intra-dermal location of the mycobacterial inoculum was validated by formation of a skin wheal at the injection site. In this experiment, *S. aureus* was included as a positive control for an organism known to infect through a superficial wound [34], [35].

**Figure 3:**
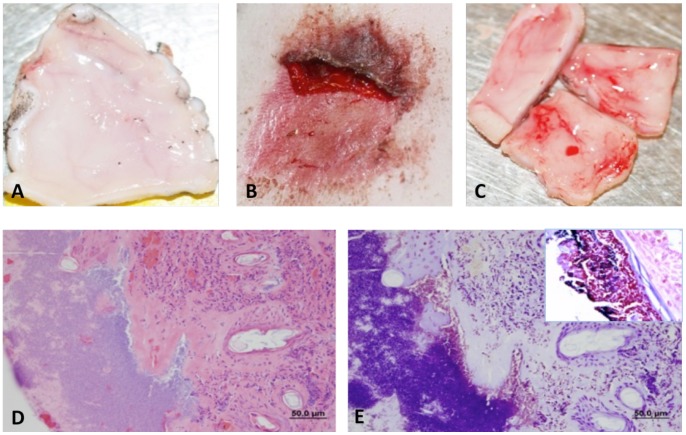
Pathology of *S. aureus* infection 24h p.i. following application to an abrasion site. **A**) Ventral side of abrasion control. **B**) Scabbing and serous exudate in a lesion following infection with *S.aureus* **C**) Ventral side of abrasion site following infection with *S. aureus* with evidence of vascularization. **D**) H&E staining of *S. aureus* lesion showing increased infiltration of inflammatory cells following *S. aureus* infection. **E**) Gram stain of *S. aureus* lesion with large accumulation of gram positive cocci associated with extracellular matrix (inset). Asterisks (*) mark location of bacteria.

Two guinea pigs infected with *S. aureus* were sacrificed 24 h p.i. The animal care and use committee suggested this short time period due to concerns regarding pain associated with *S. aureus* infection. In contrast to the abrasion control (Figure 3A), *S. aureus* infected skin showed gross pathology characterized by inflammation, scabbing and serous exudate (Figure 3B). Vascularization was also evident (Figure 3C) and histology revealed extensive infiltration of inflammatory cells (Figure 3D). Large numbers of *S. aureus* gram-positive cocci were found in association with the extracellular matrix (Figure 3E). *S. aureus* was recovered upon culture (Table 2).

**Table 2:** Detection of *M. ulcerans* and *Staphylococcus aureus* in a cutaneous infection model 1h, 24h, 48h, 7d and 14d p.i.

A. Infection with *M. ulcerans* through an open abrasion					
	Time Post Infection				
Analysis	1 Hour	24 Hours	48 Hours	7 Days	14 Days
Gross Pathology	0/2	0/2	0/2	0/2	0/2
AFB	2/2	1/2	1/2	0/2	0/2
Culture	2/2	2/2	0/2	0/2	0/2
Ave. Genome Units/Sample	2.06×10^8^	2.21×10^6^	2.69×10^6^	9.99×10^6^	Neg
	3.03×10^8^	1.15×10^7^	4.82×10^7^	5.33×10^4^	

B. Infection with *M. ulcerans* through injection					
Analysis	1 Hour	24 Hours	48 Hours	7 Days	14 Days
Gross Pathology	0/2	0/2	0/2	2/2	2/2
AFB	2/2	2/2	2/2	2/2	2/2
Culture	2/2	2/2	2/2	2/2	1/2
Ave. Genome Units/Sample	1.83×10^6^	1.53×10^6^	4.17×10^7^	3.30×10^6^	2.20×10^4^
	3.74×10^8^	6.30×10^7^	4.18×10^8^	4.02×10^8^	2.0×10^6^

C. Infection with *S. aureus* through an open abrasion					
Analysis	1 Hour	24 Hours	48 Hours	7 Days	14 Days
Gross Pathology	NA	2/2	NA	NA	NA
Gram Stain	NA	2/2	NA	NA	NA
Culture	NA	2/2	NA	NA	NA
Ave. Genome Units/Sample	NA	NA	NA	NA	NA

**Figure 4:**
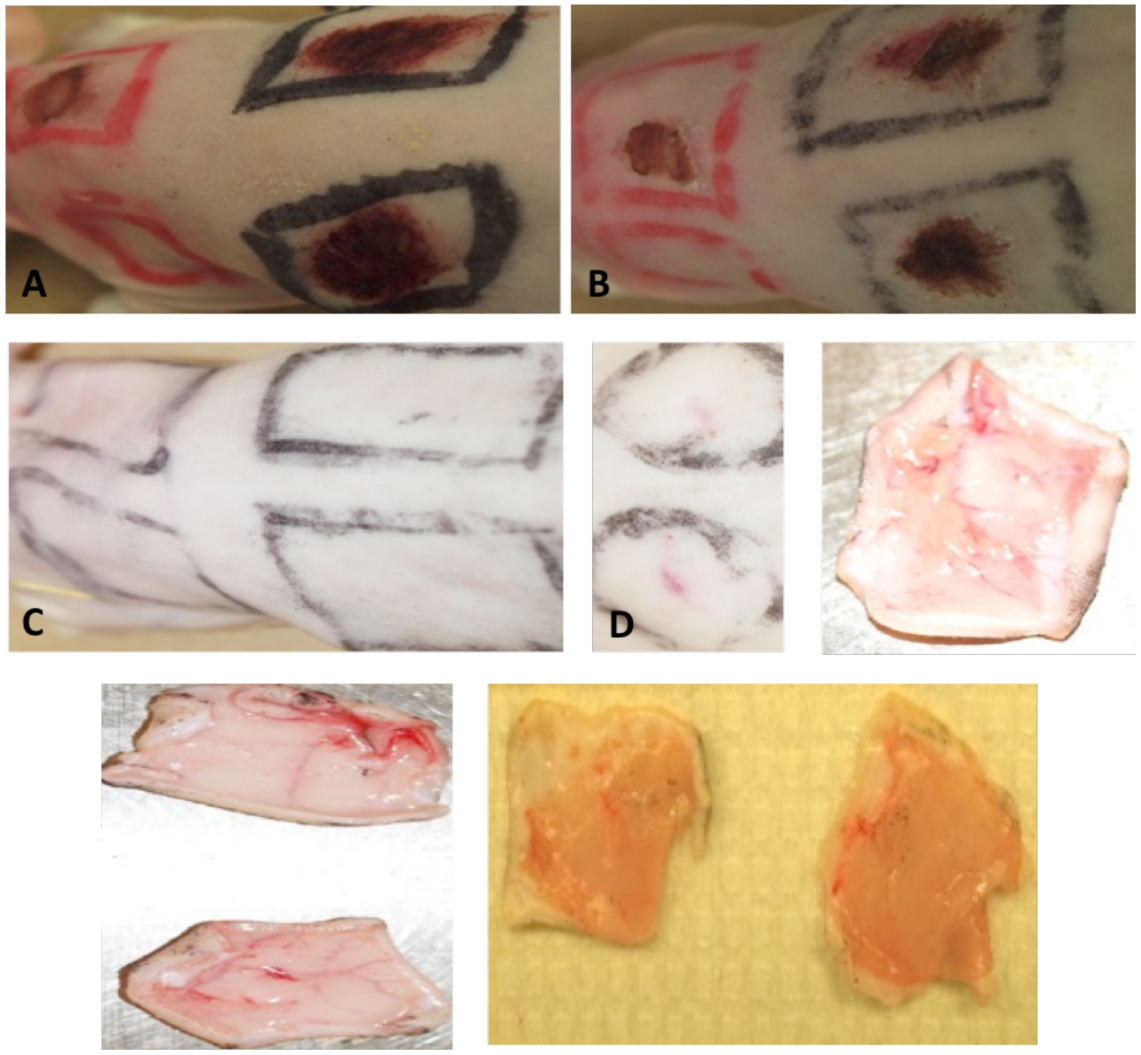
Pathology of *M. ulcerans* infection 24h, 48h, and 7d p.i. **A**) Abraded skin 24 h p.i. B) Scab formation on abrasion sites 48 hours p.i. **C**). Healed abrasion sites 14 d post infection **D**) Erythema at injection site 7 d p.i. **E**) Ventral side of abrasion control site absence of gross pathology 7 d p.i. **F**) Ventral side of abrasion site following application 10^8^ *M. ulcerans* 7 d p.i. **G**) Gross pathology of *M. ulcerans* injection site 7 d p.i. showing edema and signs of microhemorrhage.

The pathology following *M. ulcerans* infection differed greatly from that shown with *S. aureus*. Following superficial application of *M. ulcerans* to abrasions, scabs began to form by 24 h p.i. (Figure 4A), and scabs began to slough off 48 h p.i. (Figure 4B). There was no evidence of inflammation or tissue damage at the inoculated abrasion sites. All abrasions were healed within 7 d, and remained healed by the end of the 14 d study (Figure 4C). In contrast, erythema and edema were apparent at the injection site within 7 d post infection and often earlier (Figure 4D).

Gross pathology was absent at the abrasion sites where *M. ulcerans* was applied and tissue remained normal during the remainder of the 14 d study (Figure 4F). Histopathology of *M. ulcerans* infected abrasion sites was identical to that of uninfected abraded skin controls (Figure 4E). In contrast, the positive control injection site showed erythema and initial signs of typical Buruli ulcer pathology by 7 d post infection (Figure 4G) [36].

**Figure 5:**
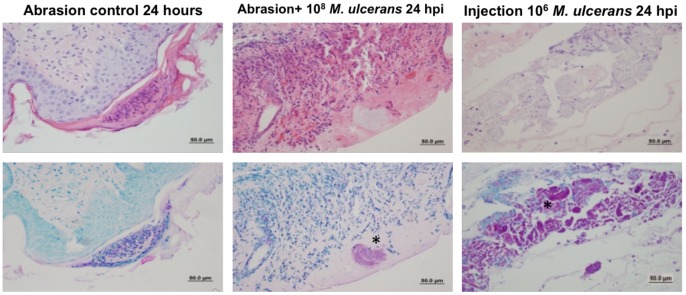
Histopathology of *M. ulcerans* infected tissues following H&E staining (top panels) and Ziehl-Neelsen staining (bottom panels). A, D ) control site where sterile media was applied to abraded skin, **B, E**) abrasion site following topical application of *M. ulcerans* to abraded skin, **C, F**) Injection site following intradermal inoculation with *M. ulcerans*. Asterisks (*) bacterial presence.

Microscopically, acid-fast bacilli were only found on sections from one of the four abrasion sites at 24 h and one of the four taken at 48 h (Table 2, Fig. 5). In both cases, a single cluster of acid-fast bacteria was found after extensive microscopic examination. These clusters did not appear to be cell associated (Figure 5E). Histology of H&E stained tissue from *M. ulcerans* infected abrasion sites (5B) was identical to that of negative controls (5A). Small numbers of *M. ulcerans* bacteria were found in clusters in abrasion sites 48 h p.i, but were absent by 7 d (Table 2). Histology of sectioned tissue from injection sites 24 h p.i. showed typical Buruli ulcer pathology (5C, 5F). Hyperplasia and infiltration of inflammatory cells were detected along with extensive necrosis of adipose tissue and micro-hemorrhage. Necrosis extended to the muscle tissue in some animals. Acid-fast staining revealed a large area of necrosis filled with large clusters of extracellular acid-fast bacilli (5F).

*Mycobacterium ulcerans* was recovered upon culture from abrasion sites at 1 h and 24 h p.i., but was not recovered at subsequent timepoints (Table 2). In contrast, *M. ulcerans* was cultured from all of the injection sites from every timepoint (Table 2).

Quantitative PCR was conducted on sectioned tissue (Table 2). *Mycobacterium ulcerans* DNA was detected from abrasion sites at 1 h p.i., with an average of 2.54×10^8^ genome units/sample. *Mycobacterium ulcerans* DNA was also detected at the 24 h, 48 h, and 7 d timepoints from abrasion sites with an average of 6.86×10^6^, 2.54×10^7^, and 5.02×10^6^ genome units/sample respectively. *Mycobacterium ulcerans* DNA was not detected from abrasion sites 14 d p.i. (Table 2) Control tissues were negative for *M. ulcerans* DNA.

*Mycobacterium ulcerans* DNA was detected at all of the injection sites throughout the study with average concentrations of 1.84×10^8^, 1.96×10^7^, 1.77×10^8^, 1.26×10^8^, and 5.79×10^6^ genome units/sample for the 1 h, 24 h, 48 h, 7 d, and 14 d timepoints respectively (Table 2).

## Discussion

In this work, we have developed an animal model to test alternative hypotheses regarding transmission of *M. ulcerans*. There is considerable controversy in the *M. ulcerans* research community regarding potential routes of transmission [1]. Whereas several publications based on both laboratory and field studies suggest that *M. ulcerans* may be transmitted through the bite of an aquatic invertebrate, vector competency studies have not been conducted [5]–[7], [15], [37], [38]. Field data are based primarily on detection of *M. ulcerans* DNA in environmental samples. Although there has been one environmental isolate from an aquatic invertebrate [38], the strain differs from those isolated in human infection, and the organism has not been isolated from a biting invertebrate. Thus, more work needs to be done to establish the role of insects in the transmission of *M. ulcerans* [1], [14].

The situation is further complicated by the following evidence: 1) *M. ulcerans* DNA has been detected in over 30 taxa of aquatic invertebrates in West Africa [11], [12], [15], [39]; and 2) none of these species are hemotaphagous, suggesting that the frequency of human bites by these insects would be extremely low. A great deal of laboratory work has been done on the interaction of *M. ulcerans* with Naucoridae [5], [40], but these species are uncommon or missing in aquatic sites sampled in Benin and Ghana [11], [14]. However, in Benin and Ghana *M. ulcerans* DNA has been repeatedly detected in Belostomatidae, a group of predatory, aquatic invertebrates, and laboratory studies confirm colonization of these insects by *M. ulcerans* both on the external skeleton and internal compartments [11], [37].

In Australia, transmission of *M. ulcerans* by mosquitoes has been proposed based on research in temperate regions of the country, but the *M. ulcerans* genome units detected in mosquitoes are extremely low making it difficult to evaluate the significance of these findings [23], [41]. Further, preliminary evidence from tropical areas of Australia where *M. ulcerans* infection occurs does not support a role for mosquitoes [42]. Laboratory studies show that whereas mosquito larvae readily consume mycobacteria, the bacteria are not maintained through pupation or adult mosquito emergence casting doubt on the role of mosquitoes as a biological vector [43]; however the potential of mosquitoes as reservoirs or their role in mechanical transmission cannot be negated.

Many investigators have suggested that *M. ulcerans* may establish infection through pre-existing wounds [1]. Although there are many types of skin lesions and wounds, the development of an abrasion model for an initial study was based on the following considerations: 1) An abrasion is a superficial lesion which does not extend below the dermis, and most dermatological bacterial pathogens such as *S. aureus* are able to establish infection through this type of minor breach in the skin; 2) Superficial skin lesions such as abrasions are ubiquitous among children in rural communities of West Africa and 3) intra-dermal injection of *M. ulcerans* also places the inocula within the dermis and this route of infection has been shown to consistently lead to Buruli ulcer [26], [28].. The fact that epidemiologic evidence fails to confirm either of these hypotheses for transmission is attributed to the highly variable and often long period of time between infection and disease [24].

Transmission of *M. ulcerans* from the environment to humans thus remains a central enigma of *M. ulcerans* research. Definitive evidence for a route of transmission could be obtained by culturing the bacteria from the environment and matching genomic data from environmental isolates with patient isolates. However, the very slow growth rate of the organism makes culture from the environment extremely difficult due to overgrowth by faster growing organisms. Despite decades of work by highly competent investigators, only one environmental isolate has been obtained and that strain was isolated from a water strider (Gerridae), an invertebrate incapable of biting humans [38]. The probability that aquatic invertebrates may serve as reservoirs, rather than vectors, *for M. ulcerans* is a strong possibility [1], [15].

When we began the investigations reported here, our hypothesis was that *M. ulcerans* could establish infection through an abrasion. Thus, the failure to establish an infection through passive inoculation was completely contrary to our expectations. Because of this surprising result, we repeated the experiment multiple times with differing amounts of inocula. Identical results were obtained in each experiment, i.e. we were unable to establish infection when *M. ulcerans* was applied to an abrasion, whereas injection of *M. ulcerans* produced an ulcer in every case. A time course over a two-week period showed evidence for transient colonization of abrasions, but after 48 h, bacteria could no longer be recovered from infected abrasions.

What might account for the inability of *M. ulcerans* to establish an infection through application to an abrasion? One intriguing possibility is that the high temperature and low oxygen environment of the injection site or the presence of fatty acids released by dead adipocytes might enhance production of the mycolactone toxin. This upregulation would clearly lead to greater pathology. Thus far, studies conducted *in vitro* show that the production of mycolactone is constitutive [44]. However, this area of investigation needs further attention.

A second possibility for the lack of colonization through an open abrasion is that *M. ulcerans* lacks adhesins for cellular proteins. In support of this hypothesis, adhesins have not been reported in *M. ulcerans*, and a search of the annotated *M. ulcerans* genome does not reveal the presence of the adhesins found in *M. marinum* or *M. tuberculosis* [45]. Evidence from histopathology shown in this paper and similar results reported from many papers on human infection describe massive clumps of *M. ulcerans* lying in necrotic tissue [46]–[48]. Early studies conducted in our laboratory with L929 and HeLa cells showed that *M. ulcerans* was unable to adhere to non-phagocytic cells (Small unpublished data). This finding is remarkable because even the saprophyte *M. smegmatis* adheres to and enters fibroblasts though replication does not occur. Data from the *M. ulcerans* genome, as well as from lipid analysis of the *M. ulcerans* surface, show that the lipid repertoire of the *M. ulcerans* cell surface is extremely small compared with other mycobacterial species [49].

*Mycobacterium ulcerans* is thought to have evolved from an *M marinum*-like ancestor through acquisition of a plasmid encoding mycolactone, and reductive evolution in which over 700 genes present in *M. marinum* are mutated or lost in *M. ulcerans* [50]. Many of these genes encode surface molecules that could play a role in bacterial-host cell interactions. An example of such a molecule would be a glycolipid present in *M. marinum* but absent from *M. ulcerans* [51]. Although both *M. ulcerans* and *M. marinum* are associated with aquatic sources, the epidemiology of the two species differs considerably. The primary risk factor for *M. marinum* infection involves handling fish, and fishing is a high-risk activity [52]. *M. marinum* has been isolated from infected fish around the world and is primarily a pathogen of aquatic vertebrates [52]. In contrast, *M. ulcerans* has not been associated with fish infection, though specific clades of *M. marinum* that have the mycolactone plasmid have caused fish infections [53]. *M. marinum* appears to be considerably more infectious than *M. ulcerans*. There have been outbreaks of *M. marinum* associated with contaminated water where dozens of people have been infected [52]. Finally, *M. marinum* appears to be able to infect skin where no apparent pre-existing lesion was noted [54]. This makes the inability of *M. ulcerans* to infect an abrasion all the more surprising.

The presence of mycolactone on the cell surface may also play a role in the failure of *M. ulcerans* to associate with cells either through its effect on the hydrophobicity of the bacterial surface, or through its activity on eukaryotic cells. This question could be addressed by comparing the ability of WT and mycolactone deficient mutants to adhere to cells. Thus, evidence from *in vivo*, *in vitro*, and *in silico* studies suggests that *M. ulcerans* is deficient in the ability to adhere to eukaryotic cells and that this defect is likely to explain the inability of *M. ulcerans* to colonize through passive inoculation of an open abrasion.

In summary, this work lends support to the hypothesis that *M. ulcerans* infection occurs through injection of bacteria rather than through entrance of pre-existing, superficial skin abrasions. The ability to establish infection through intra-dermal injection shows that inoculation does not need to be deep. In rural communities, skin wounds of many types are common. Our work does not rule out the possibility that infection could occur through puncture wounds, or lacerations. We plan to examine these possibilities more thoroughly in subsequent studies. Still, the possibility that transmission could occur through the bite of an invertebrate vector, an idea proposed by Francoise Portaels over 10 years ago, gains some support from the studies presented here.

## Supporting Information

Figure S1 **A) Experimental design with control and infection sites.** (**B**) Guinea pig 5 minutes p.i. 1) Sterile M7H9 media applied to abraded skin; 2) 10^8^ M. *ulcerans* applied to unabraded skin; 3) 10^4^ M. *ulcerans* applied to abraded skin; 4) 10^8^ M. *ulcerans* applied to abraded skin 5) 10^6^ M. *ulcerans* injected intradermally. Topical applications were applied in a 20 µL volume; injections were delivered in a 200 µL volume.
(TIFF)

Figure S2 **Quantitative PCR data from guinea pig tissue infected with** ***M. ulcerans*** **at 90d.** (**A**) Quantitative PCR graph showing cycle threshold (Ct) versus fluorescence for each sample. Dotted line indicates threshold. (**B**) Standard curve Ct versus log DNA dilution used to determine qPCR efficiency and optimization, and tissue sample results. Black dots indicate standard DNA dilutions, and gray dots indicate samples. R^2^ = 0.997, and slope = −3.1. (**C**) Individual data for guinea pig tissue. GP-1 and GP-2 indicates tissues used as controls; GP-3 and GP-4 indicates abraded skin samples where *M. ulcerans* 10^4^ or 10^8^ CFU was applied. GP-5 indicates guinea pig tissue where 10^6^ CFU *M. ulcerans* was injected intradermally.
(TIFF)
