# Supplementary figures and images for: Mycobacterium ulcerans Fails to Infect through Skin Abrasions in a Guinea Pig Infection Model: Implications for Transmission
Source: PLoS Negl Trop Dis. 2014 Apr 10;8(4):e2770. doi: 10.1371/journal.pntd.0002770 (PMC3983084; doi:10.1371/journal.pntd.0002770)

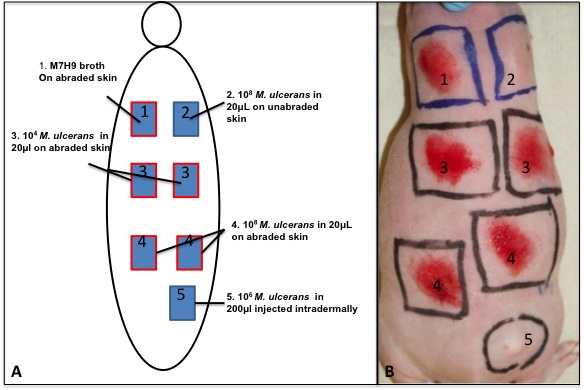

Supplement: Figure S1 — A) Experimental design with control and infection sites. (B) Guinea pig 5 minutes p.i. 1) Sterile M7H9 media applied to abraded skin; 2) 108 M. ulcerans applied to unabraded skin; 3) 104 M. ulcerans applied to abraded skin; 4) 108 M. ulcerans applied to abraded skin 5) 106 M. ulcerans injected intradermally. Topical applications were applied in a 20 µL volume; injections were delivered in a 200 µL volume. (TIFF) [file pntd.0002770.s001.tiff]

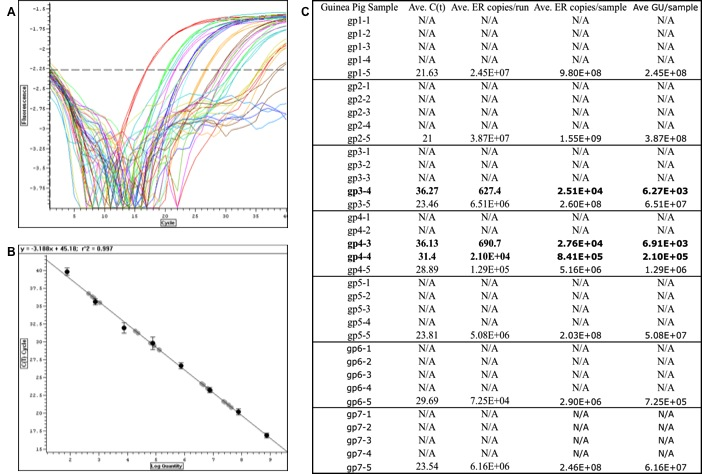

Supplement: Figure S2 — Quantitative PCR data from guinea pig tissue infected with M. ulcerans at 90d. (A) Quantitative PCR graph showing cycle threshold (Ct) versus fluorescence for each sample. Dotted line indicates threshold. (B) Standard curve Ct versus log DNA dilution used to determine qPCR efficiency and optimization, and tissue sample results. Black dots indicate standard DNA dilutions, and gray dots indicate samples. R2 = 0.997, and slope = −3.1. (C) Individual data for guinea pig tissue. GP-1 and GP-2 indicates tissues used as controls; GP-3 and GP-4 indicates abraded skin samples where M. ulcerans 104 or 108 CFU was applied. GP-5 indicates guinea pig tissue where 106 CFU M. ulcerans was injected intradermally. (TIFF) [file pntd.0002770.s002.tiff]
